# A Causal Relationship between Type 2 Diabetes and Candidiasis through Two-Sample Mendelian Randomization Analysis

**DOI:** 10.3390/microorganisms12101984

**Published:** 2024-09-30

**Authors:** Juan Xiong, Hui Lu, Yuanying Jiang

**Affiliations:** Department of Pharmacy, Shanghai Tenth People’s Hospital, School of Medicine, Tongji University, Shanghai 200092, China

**Keywords:** type 2 diabetes, candidiasis, genetic causal relationship, Mendelian randomization

## Abstract

The potential relationship between type 2 diabetes (T2D) and candidiasis is of concern due to the respective characteristics of these conditions, yet the exact causal link between the two remains uncertain and requires further investigation. In this study, the inverse-variance-weighted (IVW) analysis indicated a significant genetic causal relationship between T2D and candidiasis (*p* = 0.0264, Odds Ratio [OR], 95% confidence interval [CI] = 1.1046 [0.9096–1.2996]), T2D (wide definition) and candidiasis (*p* = 0.0031, OR 95% [CI] = 1.1562 [0.8718–1.4406]), and severe autoimmune T2D and candidiasis (*p* = 0.0041, OR 95% [CI] = 1.0559 [0.9493–1.1625]). Additionally, the MR-Egger analyses showed a significant genetic causal relationship between T2D (wide definition) and candidiasis (*p* = 0.0154, OR 95% [CI] = 1.3197 [0.7760–1.8634]). The weighted median analyses showed a significant genetic causal relationship between severe autoimmune T2D and candidiasis (*p* = 0.0285, OR 95% [CI] = 1.0554 [0.9498–1.1610]). This Mendelian randomization (MR) study provides evidence for a genetic correlation between T2D and candidiasis.

## 1. Introduction

The prevalence and fatality rates of candidiasis caused by *Candida* species have been increasing annually, resulting in significant detrimental effects to human health and imposing a substantial burden on the healthcare system [1]. Recent epidemiological data suggest that an estimated 1.56 million individuals are afflicted with candidemia or invasive candidiasis annually, leading to approximately 100,000 fatalities [2]. The complexity of treating candidiasis is attributed to the similarities in cellular characteristics between *Candida* species and human cells, thereby restricting available therapeutic interventions [3,4]. Current treatment modalities predominantly rely on azole antifungal drugs, yet these are becoming less effective as resistance and tolerance spreads [4,5,6]. Furthermore, the delay in the diagnosis of candidiasis is frequently attributed to the non-specific nature of its symptoms, allowing the pathogen to proliferate and spread within the host [7]. Consequently, there is a pressing necessity for the enhanced comprehension of the risk factors associated with candidiasis, as they may offer novel opportunities for therapeutic strategies.

Type 2 diabetes (T2D) is a metabolic syndrome characterized by inadequate insulin secretion and the reduced sensitivity of target organs to insulin, resulting in various metabolic disturbances, including disruptions in fat, protein, water, electrolyte balance, and other metabolic processes [8]. According to data from the Global Burden of Disease (GBD), the age-standardized global prevalence of T2D was approximately 6.0% in men and 5.0% in women in 2019 [9]. In T2D mellitus patients, invasive fungal disease poses a significant risk to life, primarily attributed to the pathogens *Candida albicans* (*C. albicans*), *Cryptococcus neoformans*, and *Aspergillus fumigatus* [10]. Female diabetics are often susceptible to vulvovaginal candidiasis, with *C. albicans* being the predominant species identified [11]. The occurrence and vulnerability of individuals to vulvovaginal candidiasis when linked to sodium–glucose cotransporter 2 inhibitors in clinical settings for women diagnosed with T2D were explored [12]. A case–control study involving 250 individuals with T2D and 81 nondiabetic controls in Sri Lanka revealed a notably higher prevalence of oral *Candida* colonization in diabetic patients compared to their healthy counterparts, with the coexistence of multiple yeast species being a frequent observation in the study cohort [13]. Hence, there is concern among individuals regarding T2D as a risk factor for candidiasis [14,15,16].

Investigating the causal connections between T2D and candidiasis is essential for preventing candidiasis through tailored education for individuals with T2D. Individuals with T2D face an elevated risk of developing candidiasis, influenced by factors such as hyperglycemia that can foster an environment favorable for the proliferation of *Candida* species [17]. This yeast flourishes in high-sugar conditions, and suboptimal glycemic control can amplify the risk of infection [18]. A grasp of these causal relationships enables healthcare providers to instruct patients on the significance of sustaining blood glucose levels in a healthy range to mitigate the risk of candidiasis. However, the precise causal relationship between the two conditions remains ambiguous and necessitates additional research.

Observational studies aimed at estimating causal inference are subject to various inherent limitations, including restrictions to identifying and accurately measuring confounders [19]. The Mendelian randomization (MR) research design adheres to the principles of Mendelian genetics, including specifically the random assignment of parental alleles to offspring. By positing that genotype determines phenotype, the MR method utilizes genotypes as instrumental variables (IVs) to infer associations between phenotypes and diseases [20,21]. This approach employs genetic variation as an IV to construct a model and ascertain causal effects. Currently, the MR method is extensively employed to evaluate causal relationships between traits and diseases, as well as between different diseases. In this study, we employed a two-sample MR approach to investigate the potential causal relationship between T2D and candidiasis, thereby enhancing our understanding of the prevention and treatment of candidiasis.

## 2. Materials and Methods

### 2.1. Study Design

**Figure 1:**
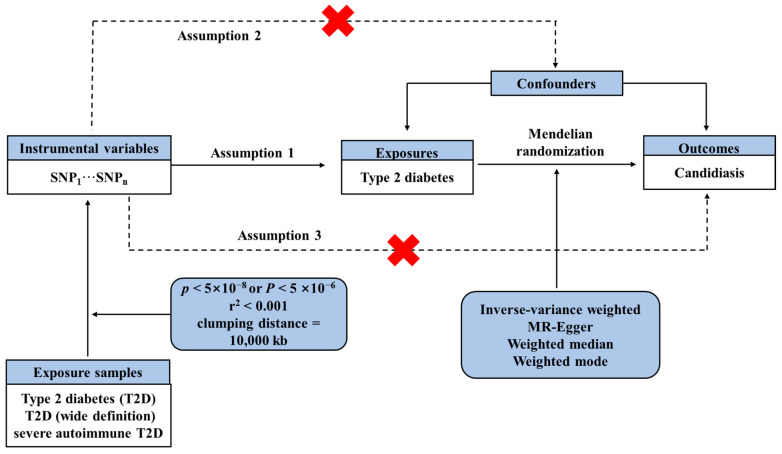
Schematic diagram illustrating the underlying assumptions of Mendelian randomization analysis and workflow in this study. The red crosses signify that the hypothesis is invalid.

In this study, we considered T2D as an exposure factor, we identified SNPs significantly associated with T2D as instrumental variables, and we evaluated the impact of these SNPs on candidiasis outcomes using two-sample MR analysis. This study adhered rigorously to the three assumptions of MR analysis: (1) ensuring that selected IVs were associated with T2D; (2) confirming that IVs were not correlated with any potential confounding factors; (3) verifying that IVs could only impact candidiasis through T2D (Figure 1). However, the testing of assumptions (2) and (3) presents challenges as they are pertinent to associations involving unidentified confounders. Consequently, we utilized the MR-Egger regression coefficient estimation method to assess the existence of a horizontal pleiotropic effect and investigated if the intercept significantly differed from zero. As this study involved a re-analysis of existing data, no additional ethical approval was required.

### 2.2. GWAS Data Sources

In this study, we selected “T2D”, “T2D (wide definition)”, and “severe autoimmune T2D” as representative forms of T2D and considered “candidiasis” as representative forms of candidiasis. T2D is a chronic metabolic disorder characterized by insulin resistance and a relative deficiency of insulin. It is marked by hyperglycemia, which occurs because the body does not effectively utilize insulin. The “wide definition” of T2D encompasses a more comprehensive understanding or classification, including not only individuals with traditional clinical diagnoses based on blood glucose levels but also those with prediabetes or at high risk of developing T2D. This wider perspective considers a range of metabolic abnormalities, such as impaired glucose tolerance and insulin resistance, which may not meet the criteria for a formal T2D diagnosis but still pose significant health risks [22]. Genome-wide association studies (GWAS) summary data for “T2D” and “severe autoimmune T2D” were obtained from the GWAS Catalog (https://www.ebi.ac.uk/gwas/) (accessed on 10 May 2024) with the study accession IDs GCST90018926 [23] and GCST90026412 [24]. The GWAS summary data for “T2D (wide definition)” and candidiasis were obtained from the FinnGen consortium (https://r10.finngen.fi/) (accessed on 10 May 2024) [22]. The study population was exclusively composed of individuals of European descent, ensuring the elimination of any potential bias caused by factors related to racial admixture. The details regarding the data sources utilized and the demographic profiles of T2D and candidiasis are presented in Table 1.

### 2.3. Selection Criteria for IVs Selection

A series of stringent quality control measures were implemented in the selection of IVs for MR analysis to adhere to the three assumptions of MR and ensure the robustness and reliability of the analysis. Firstly, SNPs associated with T2D (*p* < 5 × 10^−8^), T2D (wide definition) (*p* < 5 × 10^−6^), and severe autoimmune T2D (*p* < 5 × 10^−6^) were identified. Secondly, the linkage disequilibrium (LD) between SNPs was addressed to mitigate potential bias (r^2^ < 0.001, clumping distance = 10,000 kb). Thirdly, SNPs linked to candidiasis were excluded (*p* < 1 × 10^−5^). Fourth, F statistics were computed to assess the impact of sample overlap and weak instrument bias, with an F value below 10 indicating potential bias. To ensure a robust association with the exposure variable, SNPs with an F statistic greater than 10 were chosen as IVs [25,26]. The F statistic was calculated using the following formula: F = R^2^× (N − K − 1)/K× (1 − R^2^), where R^2^ represents the cumulative explained variance of selected SNPs on T2D, K is the number of selected SNPs, and N is the sample size. The calculation of R^2^ was performed according to the following formula: R^2^ = (2 × EAF × (1 − EAF) × Beta^2^)/[(2 × EAF × (1 − EAF) × Beta^2^) + (2 × EAF × (1 − EAF) × N × SE^2^)], where EAF denotes the effect allele frequency, Beta signifies the effect size, and SE represents the standard error of the effect size [27].

### 2.4. Mendelian Randomization Analysis

Utilizing the specified IVs, a two-sample MR analysis was conducted on the relationship between T2D and candidiasis using the TwoSampleMR package (version 0.5.5, Stephen Burgess, Chicago, IL, USA) in R (version 4.0.3). The analysis encompassed five distinct approaches: inverse-variance-weighted (IVW, random effects) as the primary method [28], supplemented by MR-Egger, the weighted median, and weighted mode. The IVW method was deemed accurate if the assumption of all included SNPs serving as effective IVs was satisfied. The MR-Egger regression method has the capability to identify and correct for pleiotropy; however, it is noted for its low estimation accuracy [29]. The weighted median method offers a precise estimation under the condition that a minimum of 50% of IVs are valid [30]. The weighted mode method is susceptible to challenges in selecting an appropriate bandwidth for mode estimation [31]. The study protocol and details were not pre-registered.

### 2.5. Sensitivity Analysis

Cochran’s Q statistic was utilized for IVW analysis to assess the heterogeneity of SNP effects on T2D-related candidiasis. A *p*-value greater than 0.05 signifies the absence of heterogeneity [32]. The intercept test of MR-Egger analysis was employed to detect potential pleiotropy and assess its impact on risk estimation in the intercept test, with a *p*-value above 0.05 indicating no pleiotropy [29]. The “Leave-one-out” analysis was conducted to examine the influence of individual SNPs on the causal relationship between T2D and candidiasis [33].

## 3. Results

### 3.1. IVs Selection

Following the removal of linkage disequilibrium (LD), a subset of 170 SNPs was found to be associated with T2D (*p* < 5 × 10^−8^) (Table S1), a subset of 84 SNPs was found to be associated with T2D (wide definition) (*p* < 5 × 10^−6^) (Table S2), and a subset of 13 SNPs was found to be associated with severe autoimmune T2D (*p* < 5 × 10^−6^) (Table S3) (Figure 1).

### 3.2. MR Analysis

**Figure 2:**
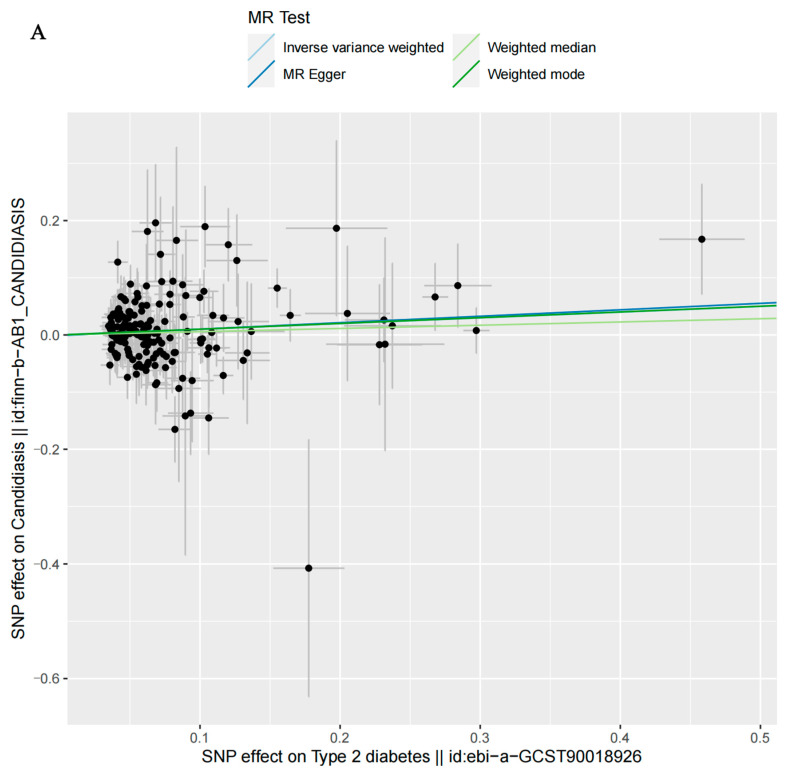

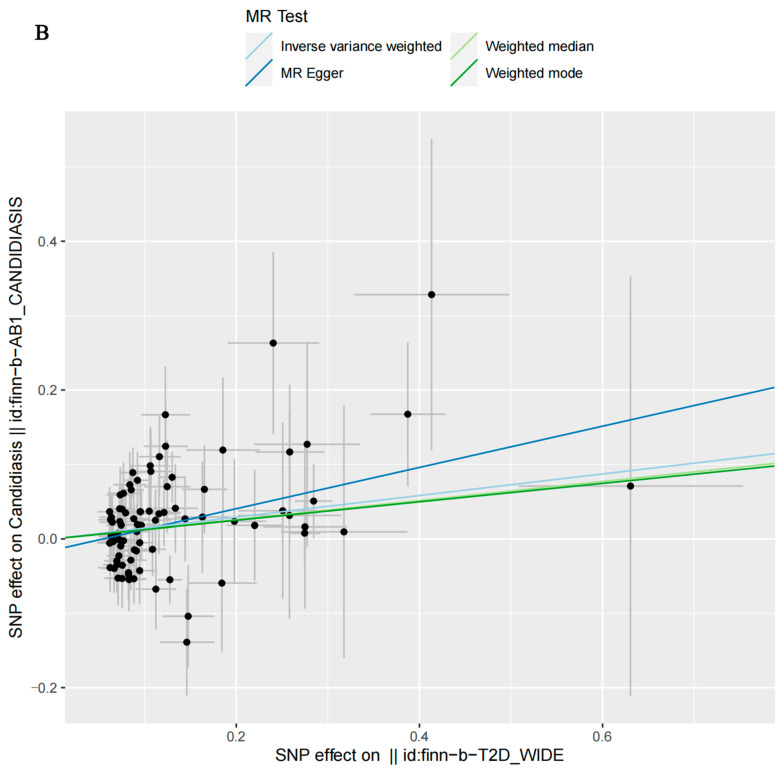

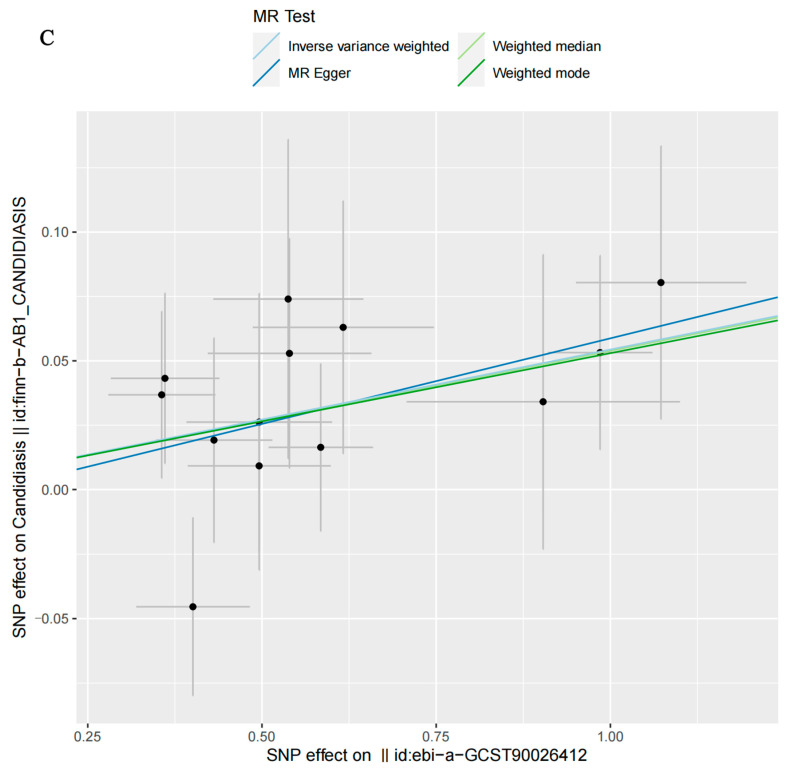
A scatter plot depicting the genetic correlations between type 2 diabetes (T2D) and candidiasis, employing various Mendelian randomization (MR) methodologies. (**A**) The causal effect estimates of T2D on candidiasis. (**B**) The causal effect estimates of T2D (wide definition) on candidiasis. (**C**) The causal effect estimates of severe autoimmune T2D on candidiasis. The slopes of the lines in the plot represent the estimated causal effects of each method. The individual SNP effects on the outcome (candidiasis) (depicted as points with vertical lines) versus the effects on exposure (T2D, T2D (wide definition), and severe autoimmune T2D (depicted as points with horizontal lines) are detailed in the background.

The MR-Egger, weighted median, and weighted mode analyses did not show a significant genetic causal relationship between T2D and candidiasis, but the IVW analysis indicated a significant genetic causal relationship between T2D and candidiasis (*p* = 0.0264, Odds Ratio [OR] 95% confidence interval [CI] = 1.1046 [0.9096–1.2996]) (Table 2). The weighted median and weighted mode analyses did not show a significant genetic causal relationship between T2D (wide definition) and candidiasis, but the IVW (*p* = 0.0031, OR 95% [CI] = 1.1562 [0.8718–1.4406]) and the MR-Egger (*p* = 0.0154, OR 95% [CI] = 1.3197 [0.7760–1.8634]) analyses indicated a significant genetic causal relationship T2D (wide definition) and candidiasis (Table 2). The MR-Egger and weighted mode analyses did not show a significant genetic causal relationship between autoimmune T2D and candidiasis, but the IVW (*p* = 0.0041, OR 95% [CI] = 1.0559 [0.9493–1.1625]) and weighted median (*p* = 0.0285, OR 95% [CI] = 1.0554 [0.9498–1.1610]) analyses indicated a significant genetic causal relationship between severe autoimmune T2D and candidiasis (Table 2). Furthermore, the beta values for the MR-Egger, IVW, weighted median, and weighted mode showed positive associations (Figure 2). These results indicate that T2D is a genetic causal risk factor for candidiasis.

There is a consensus that individuals with diabetes face a heightened risk of developing candidiasis, a condition exacerbated by elevated blood sugar levels and the subsequent pathological changes induced by hyperglycemia. Our MR study indicates a potential genetic link between T2D and candidiasis, suggesting that susceptibility to infections caused by *Candida* species in individuals with diabetes may be influenced not only by elevated blood glucose levels but also by genetic factors. Consequently, while maintaining blood sugar levels within a healthy range in diabetic patients can help decrease the incidence of candidiasis, this strategy alone may not be fully effective. This underscores the need for diabetic patients with well-managed blood sugar levels to remain vigilant for signs of candidiasis and consider drug prophylaxis when it is deemed appropriate.

### 3.3. Sensitivity Analysis Results

**Figure 3:**
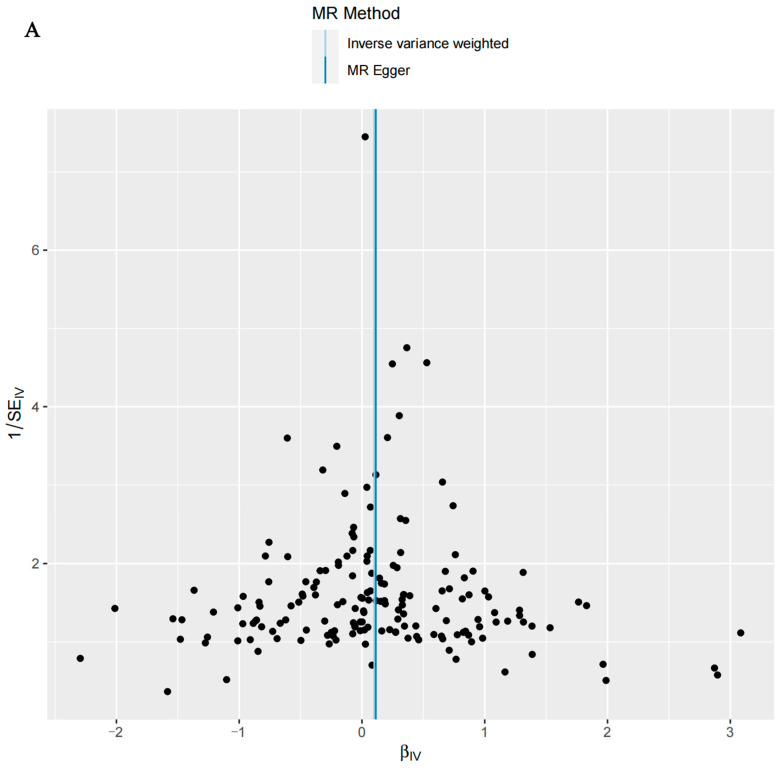

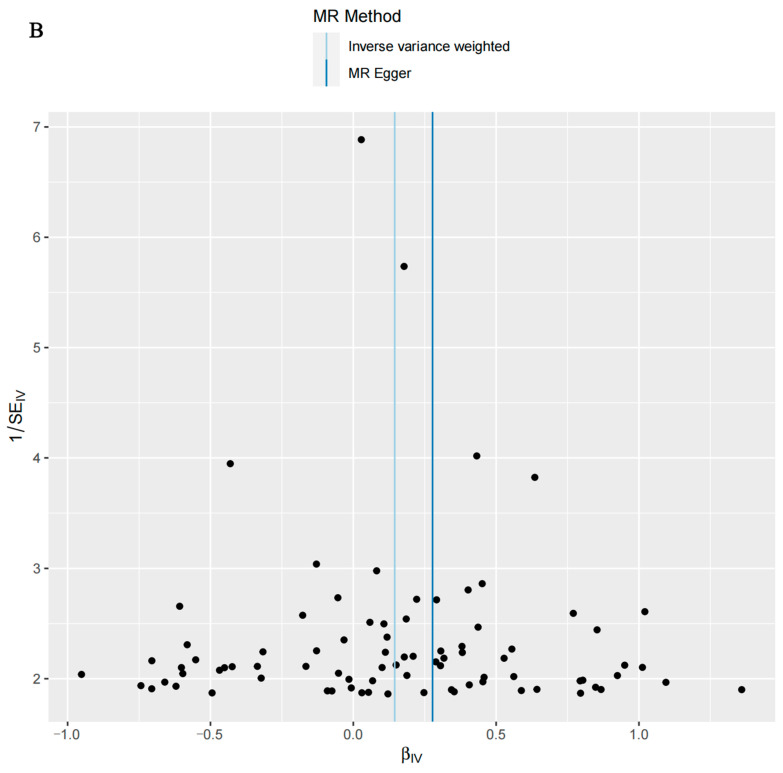

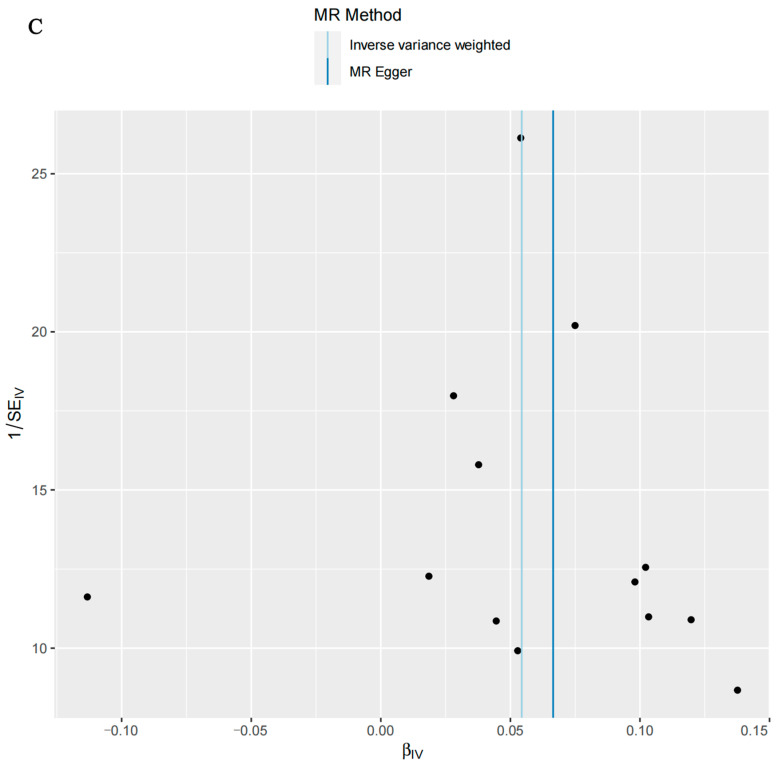
Funnel plots illustrating genetically predicted associations with (**A**) type 2 diabetes (T2D), (**B**) T2D (using a broad definition), and (**C**) severe autoimmune T2D in relation to candidiasis, employing the inverse-variance-weighted (IVW) and MR-Egger methodologies.

**Figure 4:**
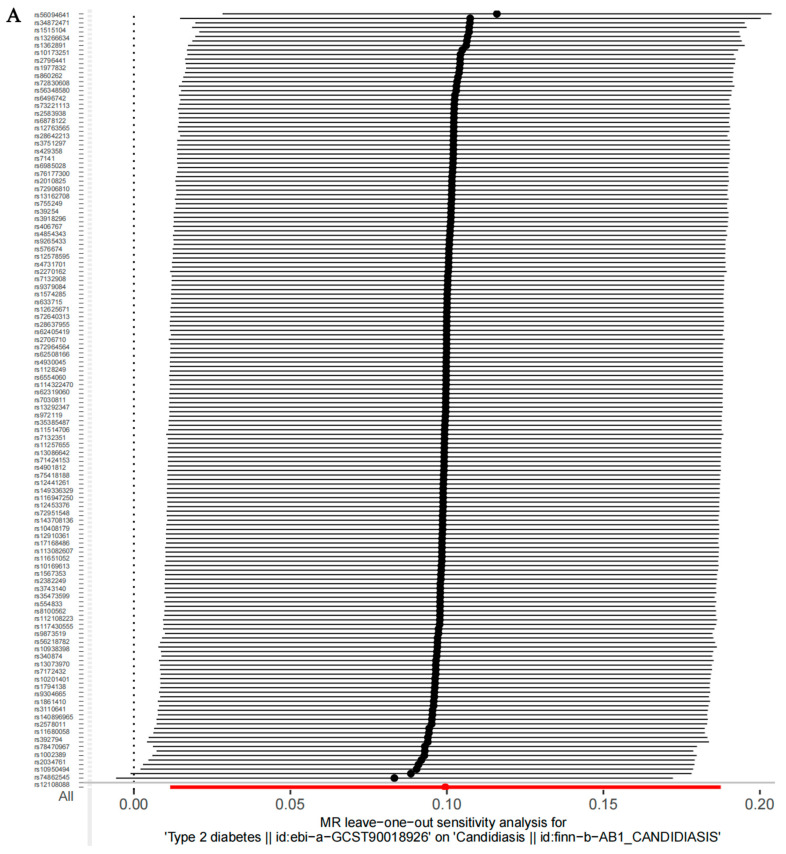

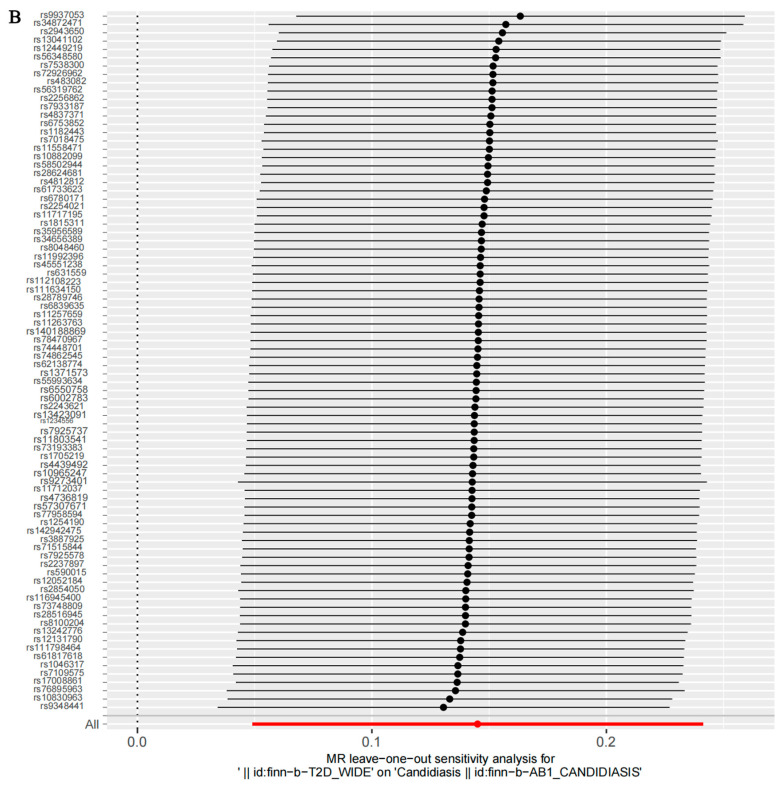

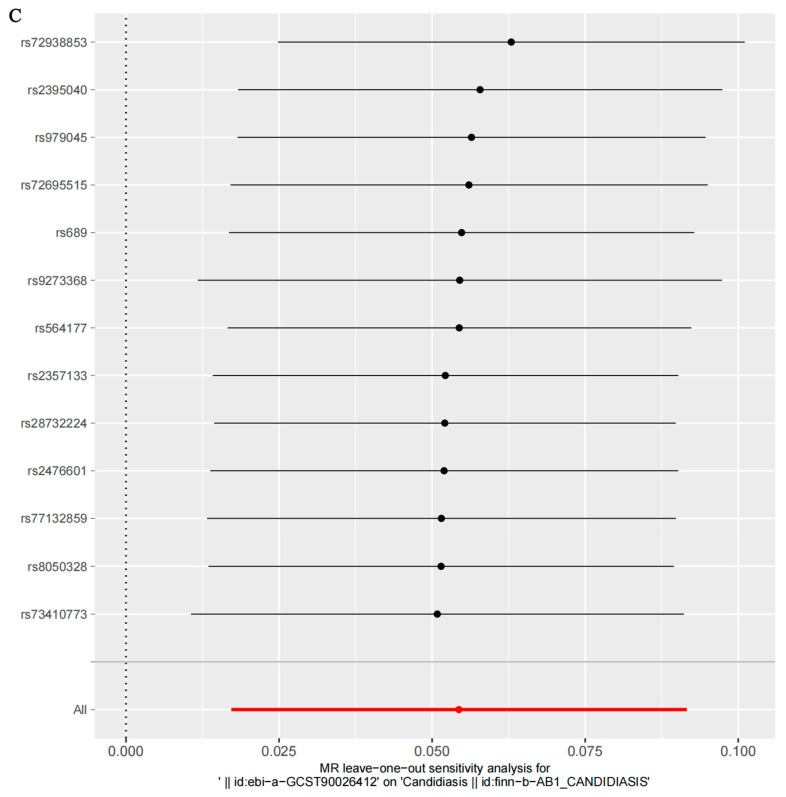
“Leave-one-out” sensitivity analysis. The MR outcomes for the remaining instrumental variables (IVs) were computed by sequentially excluding each IV. (**A**) Type 2 diabetes (T2D), (**B**) T2D (wide definition), and (**C**) severe autoimmune T2D. The red lines represent the analysis outcomes using the random effects inverse-variance-weighted (IVW) method.

Cochran’s Q statistic (IVW) indicated that there was no significant heterogeneity in the MR analyses of T2D, T2D (wide definition), and severe autoimmune T2D and candidiasis (*p* > 0.05) (Table 3). The intercept test of MR-Egger analysis further confirmed the absence of horizontal pleiotropy in the MR analyses of T2D, T2D (wide definition), and severe autoimmune T2D and candidiasis (*p* > 0.05) (Table 3). The funnel plot illustrates that the distribution of points representing causal effects is symmetrical when a single SNP is utilized as an IV, suggesting a lower susceptibility to potential bias (Figure 3). In the leave-one-out analysis, the exclusion of individual SNPs minimally impacts the results, implying that no single SNP exerts a substantial influence on the overall estimation of causal effects (Figure 4).

## 4. Discussion

This study represents the first investigation utilizing MR to examine the impact of T2D on candidiasis. Our findings establish a causal relationship between T2D and candidiasis based on the results of our MR analysis. The fundamental assumption in MR is that the IVs are linked to candidiasis solely through their association with T2D. Employing a two-sample study design, we were able to generate cost-effective and unbiased estimates to evaluate the influence of T2D on the risk of candidiasis. The F values of the independent variables suggested that they meet the strong relevance assumption of MR, with a weak instrumental bias that has minimal impact on causal effect estimates. The MR-Egger method was employed to identify and correct for pleiotropy in the genetic variants. Heterogeneity analysis showed no significant differences between SNPs, enhancing the credibility of the MR results. Our primary MR analysis utilizing SNPs associated with T2D, T2D (wide definition), and severe autoimmune T2D indicated a positive association between T2D and candidiasis according to the IVW method. Additionally, the MR-Egger analyses show a significant genetic causal relationship between T2D (wide definition) and candidiasis. The weighted median analyses showed a significant genetic causal relationship between severe autoimmune T2D and candidiasis. Consequently, we posit that T2D is a genetic risk factor for candidiasis.

*Candida* species exhibit a wide distribution across various hosts, including humans, domestic and wild animals, and diverse environments such as hospitals [34]. These species are a constituent of the typical human microflora and demonstrate the ability to colonize mucosal surfaces in areas such as the oral cavity, gastrointestinal tract, respiratory tract, and genitourinary tract [34]. Despite their commensal nature, *Candida* species have the capacity to transition from symptomless colonization to infective states [34]. The pathogenicity of *Candida* species and their colonization factors are influenced by host immune factors, establishing a complex interplay between the fungi and the host’s immune status, which ultimately determines the nature of their relationship as either commensal or parasitic [35]. The development of candidiasis in individuals with T2D is complex, with several hypotheses explaining this phenomenon. One such hypothesis posits that sustained hyperglycemia creates an ideal environment for *Candida* species by serving as a preferred energy source, thereby facilitating their survival and colonization [36]. In the context of *Candida* species infection, elevated glucose levels within infected tissues have been demonstrated to increase *Candida* species’ adherence and invasion capabilities [36,37]. Glucose also plays a role in modulating the morphological transition of *C. albicans* from yeast to hyphal forms [38], as well as in the formation of biofilms by *Candida* species cells [39]. Furthermore, the ability to detect sugars is crucial for various virulence factors, including adhesion, resistance to oxidative stress, invasion, and tolerance to antifungal medications [40]. Another hypothesis suggests that diabetes mellitus, as a prevalent endocrine disorder, enhances susceptibility to infections due to immune system impairment. This susceptibility is influenced by various factors, such as reduced T-lymphocyte counts, compromised neutrophil function, heightened leukocyte apoptosis, and diminished cytokine secretion [41]. Elevated levels of IL-10 or reduced levels of IFN-γ in individuals with diabetes may be associated with an increased risk of oral candidiasis [42]. A third hypothesis is that diabetes can induce changes in the normal microbiota, particularly in the oral and vaginal areas [43,44,45]. These changes can disrupt the equilibrium of microbial communities, allowing opportunistic pathogens like *Candida* species to proliferate. Lastly, diabetic patients are often prescribed antibiotics and steroids more frequently, which can disturb the normal microbial flora and immune function, respectively [46,47,48], fostering an environment that favors the overgrowth of *Candida* species. Upon examining these results, it becomes evident that there is a consensus that individuals with diabetes are prone to developing candidiasis due to elevated blood sugar levels and the pathological alterations that result from such hyperglycemia. According to this perspective, the risk of candidiasis can be mitigated by effectively managing the blood sugar levels of diabetic patients. Nevertheless, the extent to which the genetic background of individuals with diabetes contributes to the onset of candidiasis remains an area insufficiently explored by current research.

The MR study is an epidemiological method that employs genetic variants as tools to determine causal links between modifiable risk factors and health outcomes, addressing issues like confounding and reverse causation in observational studies. The key principles of MR include the use of genetic variants, such as SNPs, which are linked to risk factors of interest but not directly to the outcome, except through the risk factor. This approach is like random allocation in controlled trials, which helps evenly distribute confounding factors and reduce bias. By analyzing the relationship between these genetic variants and health outcomes, researchers can infer causality [20]. Our MR study indicates a potential genetic link between T2D and candidiasis, suggesting that individuals with diabetes may be more susceptible to infections caused by *Candida* species, not only because of elevated blood glucose levels and compromised immune function but also due to genetic factors. Therefore, the effective management of blood sugar levels and immune system health in diabetic patients may help reduce the likelihood of developing candidiasis, but this may not be enough on its own. This implies that diabetic patients with well-controlled blood sugar levels should remain vigilant for the development of candidiasis, and drug prophylaxis may be necessary when appropriate.

This study is subject to several limitations. Firstly, the analysis was limited to populations from Europe; therefore, caution must be exercised when generalizing the conclusions to other populations. Secondly, gender was not considered in this study, necessitating the consideration of potential differences when applying the results to male or female populations separately. Thirdly, this study exclusively examined the relationship between T2D and candidiasis without differentiating between candidiasis attributed to various *Candida* species, including *C. albicans*, *Candida parapsilosis*, and *Candida auris*. Moreover, there was no distinction made between different manifestations of candidiasis, such as oral candidiasis, vulvovaginal candidiasis, candidemia, and invasive candidiasis, among others. Lastly, the occurrence and progression of T2D is a multifaceted process, and the analysis did not consider the impact of T2D resulting from different etiologies based on susceptibility to candidiasis.

## 5. Conclusions

In conclusion, the findings from our two-sample MR analysis suggest that T2D is a substantial genetic risk factor for candidiasis. Individuals with T2D should remain vigilant for the development of candidiasis, and pharmacological interventions may be warranted to mitigate the risk of candidiasis onset.

## Figures and Tables

**Table 1:** Data sources utilized in the present study.

Exposures or Outcome	Number of Case	Number of Control	Ancestry	GWAS ID
Type 2 diabetes	38,841	451,248	European	ebi-a-GCST90018926
Type 2 diabetes (wide definition)	17,268	184,778	European	finn-b-T2D_WIDE
Severe autoimmune type 2 diabetes	452	2744	European	ebi-a-GCST90026412
Candidiasis	2015	214,816	European	finn-b-AB1_CANDIDIASIS

**Table 2:** MR results.

Exposure	Methods	No. of SNPs	*p*-Value	OR	OR_LCI95	OR_UCI95
T2D	MR Egger	170	0.2100	1.1193	0.8984	1.3402
	Weighted median	170	0.4490	1.0583	0.9473	1.1693
	Inverse-variance-weighted	170	0.0264	1.1046	0.9096	1.2996
	Weighted mode	170	0.2775	1.1057	0.9087	1.3027
T2D, wide definition	MR Egger	84	0.0154	1.3197	0.7760	1.8634
	Weighted median	84	0.0778	1.1370	0.8853	1.3887
	Inverse-variance-weighted	84	0.0031	1.1562	0.8718	1.4406
	Weighted mode	84	0.2801	1.1320	0.8890	1.3751
Severe autoimmune T2D	MR Egger	13	0.2270	1.0688	0.9384	1.1991
	Weighted median	13	0.0285	1.0554	0.9498	1.1610
	Inverse-variance-weighted	13	0.0041	1.0559	0.9493	1.1625
	Weighted mode	13	0.0984	1.0544	0.9506	1.1582

**Table 3:** MR sensitivity analyses.

Exposures	Outcomes	No. of SNPs	Cochran’s Heterogeneity Test		Pleiotropy Test	
			Single-SNP IVW		MR-Egger Intercept	
			Q	*p*-Value	Intercept	*p*-Value
T2D	Candidiasis	170	193.1	0.09842	−0.0011	0.865
T2D (wide definition)	Candidiasis	84	101.2	0.08546	−0.015	0.194
Severe autoimmune T2D	Candidiasis	13	6.423	0.8933	−0.0078	0.807

## Data Availability

The original contributions presented in the study are included in the article/Supplementary Materials, further inquiries can be directed to the corresponding authors.

## Supplementary Materials

The following supporting information can be downloaded at https://www.mdpi.com/article/10.3390/microorganisms12101984/s1, Tables S1–S3 provide a summary of SNPs associated with T2D. (T2D has a wide definition, including severe autoimmune T2D in this research).
